# Research and Design of a Bidirectional Self-Propelled Traveling Wave Type Linear Ultrasonic Motor

**DOI:** 10.3390/mi17030355

**Published:** 2026-03-13

**Authors:** Danhong Lu, Nan Sun, Yao Chen, Wenjian Qian, Xiaoxiao Dong, Bowen Chang

**Affiliations:** 1 School of Electric Power Engineering, Nanjing Institute of Technology, Nanjing 211167, China; y00450230633@njit.edu.cn (N.S.); y00450230509@njit.edu.cn (Y.C.); y00450230728@njit.edu.cn (W.Q.); 2 School of Electrical and Power Engineering, Hohai University, Nanjing 210024, China; dongxiaoxiao@hhu.edu.cn; 3 Engineering Training Center, School of Applied Technology, Nanjing Institute of Technology, Nanjing 211167, China; x00234220205@njit.edu.cn

**Keywords:** linear ultrasonic motor, traveling wave, piezoelectric ceramic, finite element analysis

## Abstract

This paper proposes a bidirectional self-propelled traveling wave linear ultrasonic motor. The motor adopts a straight-beam stator structure, with two piezoelectric ceramic plates arranged at each end of the stator. One end operates in the inverse piezoelectric mode, while the other operates in the piezoelectric mode. By switching the piezoelectric/inverse piezoelectric modes at both ends, the propagation direction of the traveling wave component in the stator can be altered, thereby achieving bidirectional operation of the linear ultrasonic motor. A finite element model of the motor is established, and its performance is analyzed through modal analysis, harmonic response analysis, and transient analysis, verifying the correctness of the design.

## 1. Introduction

Traveling-wave ultrasonic motors are advanced actuators that utilize traveling waves excited by piezoelectric ceramics and convert microscopic vibrations into macroscopic linear motion through frictional interaction [1,2,3,4,5]. According to the structural configuration of the stator, traveling-wave ultrasonic motors are generally classified into circular and linear types. Owing to their compact structure [6], fast dynamic response [7], and high positioning accuracy [8], linear ultrasonic motors have been widely applied in high-end fields such as precision instruments [9], aerospace engineering [10,11], and biomedical devices [12,13]. Notably, their high positioning accuracy and anti-electromagnetic interference make them ideal for microscopy stages, where precise motion control of the platform is critical [14]. They also show great potential in cryogenic environments for aerospace applications, as the piezoelectric materials can maintain stable performance under extreme low temperatures [15].

Despite these advantages, conventional traveling-wave linear ultrasonic motors often rely on fixed boundary conditions and complex energy-absorbing systems to generate stable traveling waves [16,17,18,19]. Such auxiliary structures not only increase the overall system size but also limit motion flexibility. To obtain high-quality traveling waves under a simplified configuration, impedance matching and appropriate vibration-mode selection are crucial. Although various approaches combining standing-wave and traveling-wave components have been investigated, achieving bidirectional self-propelled operation with a streamlined excitation/energy-absorption system remains a major challenge.

In recent years, scholars at home and abroad have made many advances in the research of traveling-wave linear ultrasonic motors. For example, in 2021, a team from Chung Yuan Christian University in Taiwan designed a short-beam linear traveling-wave ultrasonic motor [20], which consists of two sets of ceramic actuators spaced ¼ wavelength apart. It utilizes the superposition of standing waves to form a traveling wave, and by adding vibration-absorbing structures at both ends of the beam, the wave reflection is effectively reduced, achieving an output speed of 169 mm/s. In addition, addressing the demand for large thrust, Wu Jiang et al. from Shandong University proposed a self-moving ultrasonic motor driven by longitudinal traveling waves in 2022 [21]. Adopting a structure where double longitudinal transducers are connected in series on a rectangular rod vibrating body, the load-carrying and traction capabilities of the motor were significantly improved, with a maximum traction force reaching 24.5 N. Regarding miniaturization and bidirectional motion control, Spanish scholar Jorge Hernando-García designed a miniature robot driven by linear traveling waves in 2020 [22]. By changing the phase difference of the voltage applied to the piezoelectric ceramics at both ends, the switching of the motion direction was realized, and high load capacity was achieved using 3D-printed rigid driving feet.

Despite the aforementioned advances, developing a traveling-wave linear ultrasonic motor that is compact, self-propelled, features a simplified excitation/energy-absorption configuration, and can effectively suppress boundary reflections remains an urgent engineering challenge. To address the above issues, this study designs and investigates a novel bidirectional self-propelled traveling-wave linear ultrasonic motor based on the shear vibration mode (d_15_) of piezoelectric ceramics and impedance-matching energy-absorbing technology [23,24,25,26]. Excitation and energy-absorbing piezoelectric ceramics are arranged at the two ends of the stator, utilizing protruding structures at the ends for fixation and energy exchange, eliminating the need for external fixing devices. By introducing an external impedance-matching energy-absorbing circuit and switching the roles of the excitation end and the energy-absorbing end, reversible control of the traveling-wave propagation direction is achieved, enabling bidirectional motion of the motor. Meanwhile, the stator structure itself realizes self-propelled motion during operation, which significantly simplifies the mechanical structure and enhances system integration. Furthermore, through rational structural design and impedance parameter matching, an impedance-based control strategy for regulating speed and output performance is proposed, allowing the motor to achieve excellent bidirectional motion capability and speed regulation performance while maintaining a compact structure.

To verify the feasibility and performance of the proposed bidirectional self-propelled traveling-wave linear ultrasonic motor, a systematic study is carried out in this paper. First, the stator structure and material selection are optimized: multiple non-uniform protruding structures are introduced on the stator, and the difference in protrusion lengths between the middle section and the end sections is utilized to optimize the mode-shape distribution. Excitation/energy-absorbing piezoelectric ceramics are embedded at both ends of the stator, and both the metal elastomer and the driving feet are made of 7xxx-series aluminum alloy. Second, an equivalent circuit model of the motor is established, and a series impedance-matching energy-absorbing circuit is designed to suppress reflected traveling waves, increase the traveling-wave proportion, and realize speed and blocking-force regulation through impedance parameters. Then, finite element simulations, including modal analysis, harmonic response analysis, and elliptical motion trajectory analysis of the driving feet, are conducted to verify the formation of traveling waves and motion characteristics. Finally, systematic experimental tests—including voltage–speed, resistance–speed, voltage–blocking-force, and resistance–blocking-force characteristics—are performed on a dedicated experimental platform to verify the motor’s bidirectional walking and speed regulation performance.

## 2. Linear Ultrasonic Motor Structure and Principle

### 2.1. Stator Structure of the Motor

**Figure 1:**
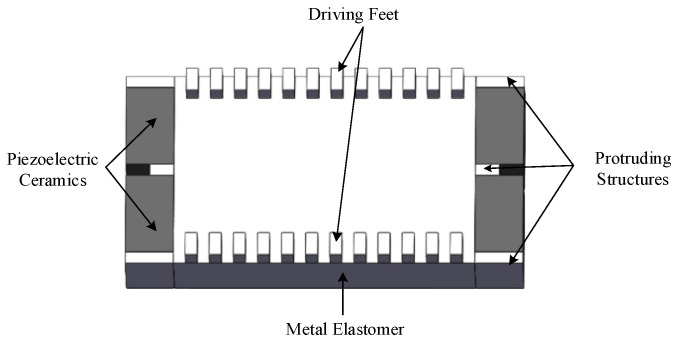
Structure of the ultrasonic motor.

The structure of the bidirectional self-propelled traveling wave linear ultrasonic motor designed in this study is shown in Figure 1. The motor mainly consists of a stator and an energy-absorbing circuit. The stator comprises a metallic elastic body, excitation/energy-absorbing piezoelectric ceramics, and driving feet. Protruding structures are arranged at the front, middle, and rear sections of the ends of the metal elastomer, with piezoelectric ceramics bonded between these protrusions. All four piezoelectric ceramics share the same polarization direction perpendicular to the stator surface. Driving feet are arranged at the bottom of the stator to achieve greater vibration amplitude. The advantage of this configuration is that the piezoelectric ceramics are embedded within the protruding end structures, thereby eliminating the external clamping fixtures typically required by conventional linear ultrasonic motors.

### 2.2. Structural Parameters of the Motor

To ensure that the desired traveling-wave bending mode is excited near the target frequency while also considering manufacturability and structural stiffness, the geometric dimensions and material parameters of the stator are carefully designed and selected in this study. The dimensions of the protruding structures in the middle and at both ends of the stator have a significant influence on the modal frequencies and mode shape distribution. The lengths of the left and right middle-end protruding structures are both 2 mm, whereas the protruding structures at the left front, left rear, right front, and right rear ends are each 4 mm. Through this non-uniform distribution, the mode shape can be optimized under the constraint of structural symmetry, enabling larger vibration amplitudes in the vicinity of the driving feet.

Both the metal elastomer and the driving feet are made of 7xxx-series aluminum alloy supplied by Henan Mingtai Al. Industrial Co., Ltd., Zhengzhou, Henan, China, with a mass density of *ρ* = 2700 kg/m^3^, Young’s modulus *E* = 7 × 10^10^ N/m^2^, and Poisson’s ratio *σ* = 0.34. This material has a relatively low density and a high specific stiffness, which not only ensures sufficient stiffness and strength of the stator, but also helps to increase the operating frequency and reduce the overall mass. Therefore, 7-series aluminum alloy is a suitable choice for applications where lightweight design and fast dynamic response are required.

The piezoelectric ceramics are made of PZT-4, with a mass density of *ρ* = 7600 kg/m^3^ and relative permittivities of *ζ_tr_*_1_ = 1300 and *ζ_tr_*_3_ = 1000 under free conditions. This material offers a high electromechanical coupling coefficient and good stability, making it well suited for ultrasonic drive applications involving relatively high frequencies and stable operating environments. By properly designing the dimensions, polarization direction, and electrode configuration of the piezoelectric ceramics, the desired bending vibration mode can be excited in the stator, thereby generating a traveling wave that propagates along the stator length. The specific structural parameters of the motor are listed in Table 1.

### 2.3. Working Principle of the Motor

**Figure 2:**
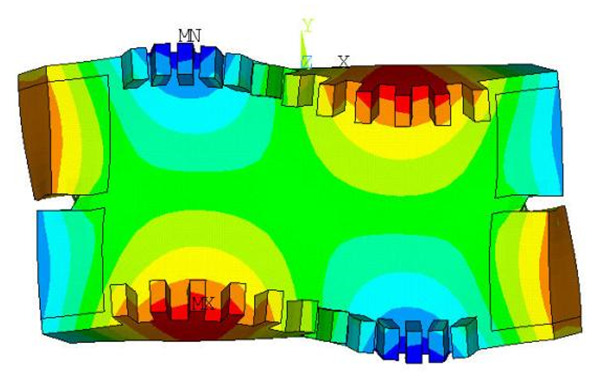
Working mode of the stator in the bidirectional self-propelled traveling wave linear ultrasonic motor.

The bidirectional self-propelled traveling wave linear ultrasonic motor designed in this paper operates in the B(3,1) mode, as shown in Figure 2. In this working mode, the bending vibrations at the front and rear sides of the stator are 180° out of phase in time.

**Figure 3:**
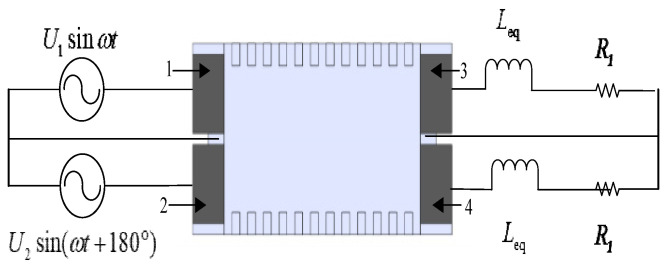
Schematic Diagram of Motor Rightward Motion.

Taking the case of the motor moving to the right as an example, two piezoelectric ceramics (PZTs) at the left end of the stator (excitation end) are driven by two high-frequency AC voltages with a phase difference of 180° and the same amplitude, as shown in Figure 3.

The piezoelectric ceramics operate in the d15 shear vibration mode, causing the protruding structure at the left end to undergo alternating up-and-down motion. This motion of the protruding structure further drives the elastic body to bend. Due to the 180° phase difference between the AC voltages applied to the two excitation-end piezoelectric ceramics, the thin-plate metallic elastic body generates bending vibrations at the front and rear sides that have the same mode shape but are 180° out of phase in time. As a result, an incident traveling wave component is formed within the elastic body. Taking the front-side incident traveling wave component as an example, its expression is:

(1) $$Y_{1}(l,t)=\xi_{1}\cos(\omega t-kl+\theta_{1})$$

where $\xi_{1}$ is the amplitude of the incident traveling wave, ω is the angular velocity of the applied electric field, k is the wavenumber ($k=\frac{2\pi }{\lambda }$), l is the wavelength of the traveling wave expressed in angular terms, t is time, and $\theta_{1}$ is the phase of the incident traveling wave.

When the incident traveling wave propagates to the opposite end of the stator (energy-absorbing end), it generates reflected and refracted traveling wave components. The waveform inside the stator is a composite wave formed by the superposition of the incident and reflected traveling wave components, and its expression is:

(2) $$Y(l,t)=Y_{1}(l,t)+Y_{2}(l,t)$$

where $Y_{2}(l,t)$ represent the reflected traveling wave component, and its expression is:

(3) $$Y_{2}(l,t)=\xi_{2}\cos(\omega t+kl+\theta_{2})$$

where $\xi_{2}$ is the amplitude of the reflected traveling wave and $\theta_{2}$ is the phase of the reflected traveling wave.

Therefore:

(4) $$\begin{array}{l} Y(l,t)\;\;\;=(\xi_{1}-\xi_{2})\cos(\omega t-kl+\theta_{1})+\\ 2\xi_{2}\cos(\omega t+\frac{\theta_{2}-\theta_{1}}{2})\cos(kl+\frac{\theta_{2}+\theta_{1}}{2}) \end{array}$$

This composite waveform contains both traveling wave and standing wave components, where the traveling wave component is:

(5) $$Y_{t}(l,t)=(\xi_{1}-\xi_{2})\cos(\omega t-kl+\theta_{1})$$

**Figure 4:**
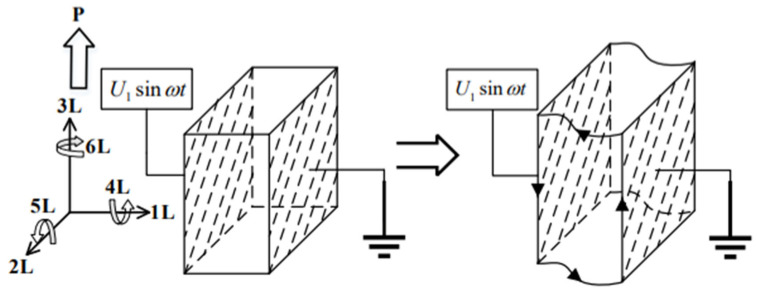
Schematic diagram of d_15_ shear vibration.

This traveling-wave component enables self-propelled rightward motion of the motor. The standing wave component is:

(6) $$Y_{s}(l,t)=2\xi_{2}\cos(\omega t+\frac{\theta_{2}-\theta_{1}}{2})\cos(kl+\frac{\theta_{2}+\theta_{1}}{2})$$

when the energy is absorbed at the left end of the stator and excitation is applied at the right end, the moving direction of the motor will change from rightward to leftward. Regardless of whether the piezoelectric ceramic operates under the piezoelectric effect or the inverse piezoelectric effect, it works in the d_15_ shear vibration mode. The silver-plated surface of the piezoelectric ceramic is parallel to the polarization direction, and the electric field direction is perpendicular to the polarization direction, as shown in Figure 4.

### 2.4. Design and Analysis of the Impedance-Matching Energy-Absorbing Circuit

**Figure 5:**
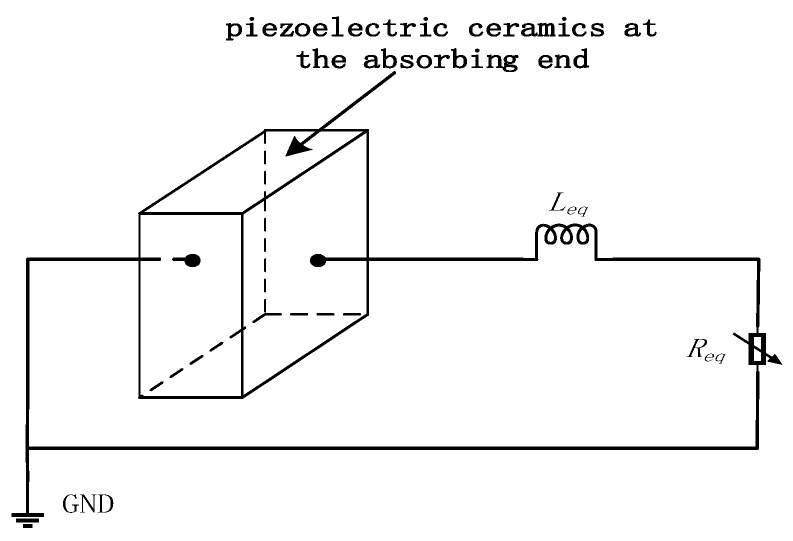
Energy-absorbing circuit.

Reducing the amplitude of the reflected traveling wave can increase the traveling wave component in the stator. To achieve this, an energy-absorbing circuit is installed at the right end of the stator, as shown in Figure 5. Through this circuit, the incident traveling wave induces the two piezoelectric ceramics at the absorbing end to operate in the d_15_ shear mode. Owing to the direct piezoelectric effect, an alternating electric field is generated on both sides of the two piezoelectric ceramics at the absorbing end. By connecting an external impedance-matching energy-absorbing circuit, the generated electrical energy is dissipated, thereby reducing the reflected traveling wave component.

Using the equivalent circuit method to transform the vibration governing equations of the ultrasonic motor into electrical equations is an effective approach for studying the electromechanical coupling behavior of piezoelectric ceramics. Under specific operating conditions, parameters such as the equivalent mass and modal stiffness of the ultrasonic motor can be determined, and the vibration of the elastomer can be converted into an equivalent circuit model for analysis.

The vibration equation derived from vibration theory is given by:

(7) $$Mq^{\prime \prime }(t)+\eta q^{\prime }(t)+K_{i}q(t)=F_{D}$$

where M is the mass of the stator, η is the viscous damping coefficient, $K_{i}$ is the modal stiffness, and $F_{D}$ is the driving force.

Equation (7) can be rewritten in the following form:

(8) $$(j\omega M+\eta +\frac{K_{i}}{j\omega })q^{\prime }(t)=F_{D}$$

where ω is the angular frequency of $F_{D}$.

Therefore, the equivalent impedance of the ultrasonic motor can generally be expressed as: $Z_{\mathrm{stator}}=j\omega M+\eta +\frac{K_{i}}{j\omega }$.

Thus, the equivalent impedance of the ultrasonic motor can be regarded as a series connection of an inductance, a resistance, and a capacitance. Let $L_{s}=M$, $R_{s}=\eta$ and $C_{s}=\frac{1}{Ki}$, then the equivalent impedance of the ultrasonic motor can be rewritten as $Z_{\mathrm{stator}}=j\omega L_{s}+R_{s}+\frac{1}{j\omega C_{s}}$, where $L_{s}$ is the equivalent inductance, $R_{s}$ is the equivalent resistance, and $C_{s}$ is the equivalent capacitance.

**Figure 6:**
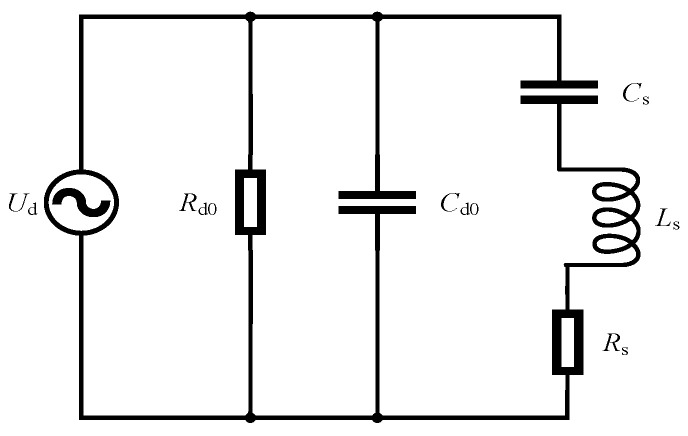
Equivalent model of the ultrasonic motor.

**Figure 7:**
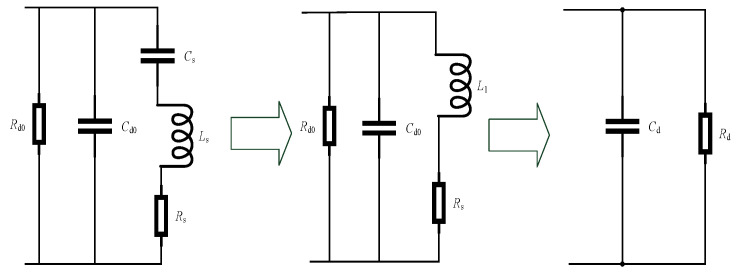
Optimized equivalent circuit model of the ultrasonic motor.

Therefore, when the ultrasonic motor operates at its resonant frequency, it can be represented by the equivalent circuit shown in Figure 6:

For convenience in analyzing the circuit, the equivalent circuit is transformed into an RC parallel circuit, as shown in Figure 7:

(9) $$L_{1}=L_{s}-\frac{1}{\omega^{2}C_{s}}$$

(10) $$C_{d}=C_{d0}-\frac{L1}{R_{s}^{2}+{\left(\omega L1\right)}^{2}}$$

where $C_{d0}$ is the clamping capacitance caused by dielectric properties.

(11) $$R_{d}=R_{d0}-\frac{R_{d0}^{2}R_{s}}{R_{d0}R_{s}+R_{s}^{2}+{\left(\omega L_{1}\right)}^{2}}$$

where $R_{d0}$ is the equivalent resistance representing dielectric loss.

**Figure 8:**
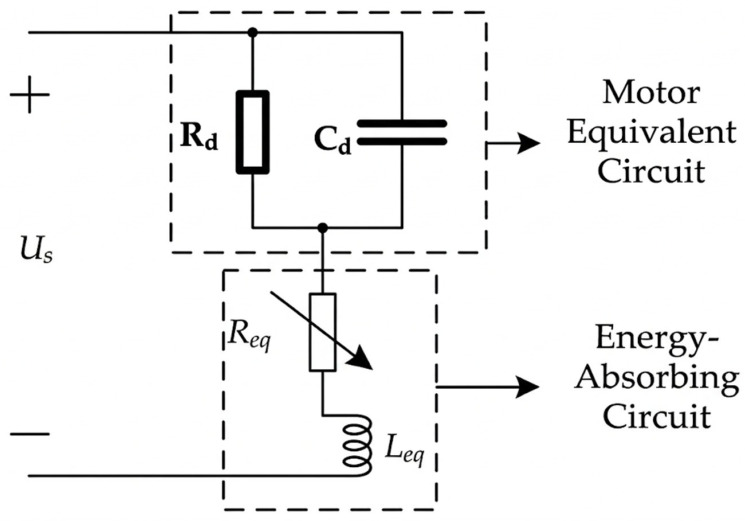
Schematic diagram of impedance matching for a bidirectional self-propelled traveling-wave linear ultrasonic motor.

For experimental convenience, and because series matching provides a better electromechanical coupling coefficient, a series impedance circuit is employed in this study to achieve impedance matching and thereby realize energy absorption. Let the resistance in the impedance-matching circuit be *R*_eq_ and the inductance be *L*_eq_. Under the excitation of a high-frequency AC power supply, an appropriate inductance is selected so that the inductive reactance equals the capacitive reactance, achieving impedance matching. The schematic diagram of the equivalent circuit of the bidirectional self-propelled traveling-wave linear ultrasonic motor with the impedance-matching energy-absorbing circuit is shown in Figure 8.

The input impedance of the ultrasonic motor is:

(12) $$Z_{\mathrm{eq}}=\frac{R_{d}}{1+R_{d}^{2}\omega^{2}C_{d}^{2}}+j(\omega L-\frac{R_{d}^{2}\omega C_{d}^{2}}{1+R_{d}^{2}\omega^{2}C_{d}^{2}})$$

Assuming the motor exhibits purely resistive behavior, the resonance condition is:

(13) $$\omega L=\frac{R_{d}^{2}\omega C_{d}^{2}}{1+R_{d}^{2}\omega^{2}C_{d}^{2}}$$

Then, the matching inductance is:

(14) $$L_{\mathrm{eq}}=\frac{R_{d}^{2}C_{d}}{1+R_{d}^{2}\omega^{2}C_{d}^{2}}$$

The matching resistance is:

(15) $$R_{\mathrm{eq}}=\frac{R_{d}}{1+R_{d}^{2}\omega^{2}C_{d}^{2}}$$

The power absorbed by the circuit at this condition is:

(16) $$P=\frac{U_{d}^{2}}{4R_{\mathrm{eq}}}$$

Therefore, when the resistance is either too high or too low, the energy-absorbing effect of the matching circuit is reduced, which increases the reflected wave component and decreases the proportion of the traveling wave, thereby increasing the standing wave ratio. As a result, the amplitudes of the two rows of driving teeth vary. Consequently, by adjusting the resistance parameter, the proportion of the traveling wave component within the motor can be controlled, enabling regulation of the motor’s speed and blocking force, as well as the realization of directional motion.

## 3. Finite Element Analysis

**Figure 9:**
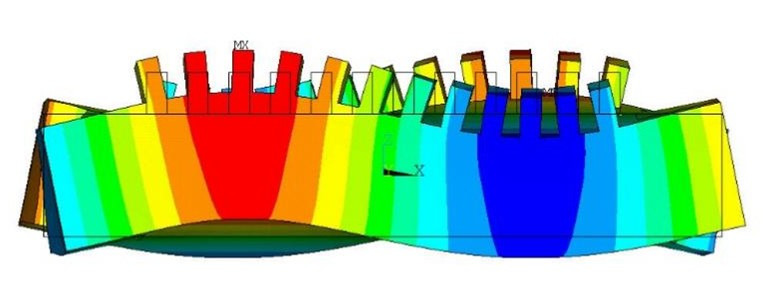
Vibration modes under harmonic response of the bidirectional self-propelled traveling-wave linear ultrasonic motor.

A harmonic response analysis of the motor model was performed, as illustrated in Figure 9. The structural parameters of the motor are listed in Table 1. The excitation frequency was set to 68.698 kHz, the piezoelectric ceramic material used was PZT4, and the inductance and resistance values of the energy-absorbing circuit at the absorbing end were 32.5 mH and 400 Ω, respectively.

**Figure 10:**
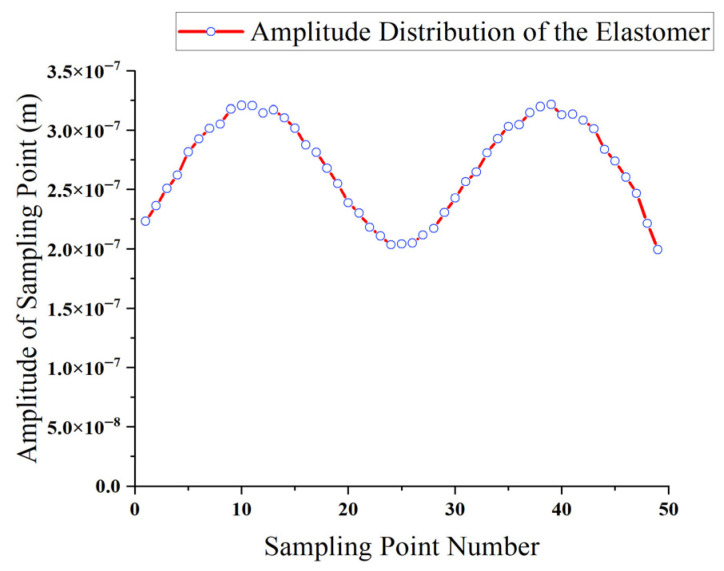
Amplitude distribution of the bidirectional self-propelled traveling-wave linear ultrasonic motor.

Since the stator waveform comprises both traveling wave and standing wave components, the amplitudes of the driving feet vary at different locations of the motor. The amplitudes of 50 nodes were extracted from the front surface of the stator. The maximum node amplitude was 0.341 μm, and the minimum amplitude was 0.211 μm. The amplitude distribution is shown in Figure 10.

As shown in Figure 10, the vibration amplitudes vary across different positions of the stator. The amplitude distribution exhibits two wave crests and one wave trough, which is attributed to the presence of standing wave components within the stator. The difference between the maximum and minimum amplitudes is 0.13 μm, indicating that the stator contains a significant traveling wave component and a relatively small standing wave component. This demonstrates the effectiveness of the motor’s excitation and energy absorption configuration.

**Figure 11:**
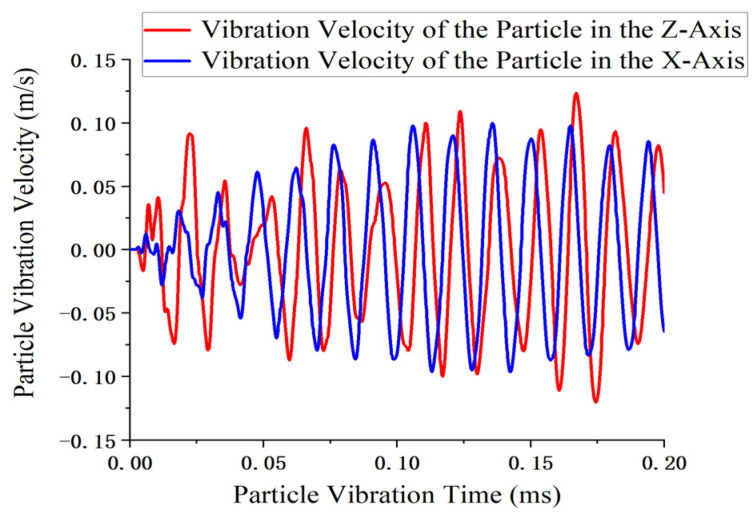
Transient displacement response of the particle.

After confirming the vibration mode, resonant frequency, impedance matching parameters, and calculating the key performance indicators of the motor, it is necessary to analyze the motion trajectory of the driving tooth to verify whether it conforms to the initially designed elliptical trajectory. To this end, a transient analysis was conducted to determine the trajectory of the driving tooth. To observe the results in the transient analysis, Node 6932 was randomly selected as the observation point. Post-processing was performed on the displacement results of this node under all load steps to determine the time-dependent variation in the displacement. The transient displacement response of the particle, as calculated from the analysis, is shown in Figure 11.

According to the results shown in Figure 11, the ultrasonic motor designed in this study exhibits a rapid dynamic response, with vibration at node 6932 initiated 0.146 μs after power is applied. The maximum displacement amplitude in the Z-direction reaches 0.296 μm, while that in the X-direction reaches 0.228 μm.

**Figure 12:**
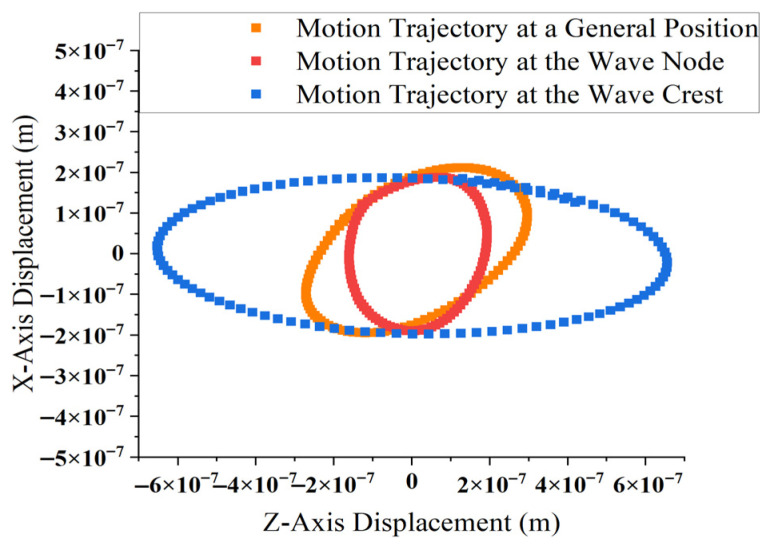
Motion trajectories of the driving tooth tip at wave crest, wave node, and general positions.

Based on the concepts of standing wave crest and node, the position with the maximum amplitude is defined as the wave crest location of the composite waveform Y(l,t) , and the position with the minimum amplitude is defined as the wave node location Y(l,t). To analyze the variation in motion characteristics, the trajectories of the driving tooth tip are examined at three representative positions along the stator’s elastic body: the wave crest, wave node, and an intermediate position. At 0.1 m s, the Z-direction and X-direction displacements of the driving tooth tip particles at these three positions are extracted and plotted within the same coordinate system. The resulting motion trajectories at the crest, node, and general positions are shown in Figure 12.

According to Figure 12, at the general position of the composite waveform, the particle’s trajectory is an inclined ellipse. At the wave node position, the trajectory approaches a near-perfect ellipse, and similarly, at the wave crest position, the trajectory also approximates a regular ellipse. These results further confirm that the motion trajectory of the driving tooth in the proposed bidirectional self-propelled traveling wave linear ultrasonic motor aligns well with the expected elliptical trajectory, thereby validating the rationality of the ’motor’s structural design and operational characteristics.

## 4. Experiment

### 4.1. Introduction to the Experimental Platform

To verify the structural rationality and motion performance of the proposed bidirectional self-propelled traveling-wave linear ultrasonic motor, an experimental test platform was established to investigate the motor velocity characteristics and performance parameters.

The experimental platform mainly consists of three parts: the driving circuit, the motor fixture system, and the measurement instruments.

The driving circuit includes a Signal Generator, DC Power Supply, Power Amplifier Circuit, and Transformer, which are used to generate and amplify high-frequency excitation signals for the ultrasonic motor.

The motor fixture system comprises the Bidirectional Self- Propelled Traveling Wave Linear Ultrasonic Motor, Impedance-Matching Energy-Absorbing Circuit, Motor Fixture Structure, Mover, and Base, providing stable support and proper boundary conditions for motor operation.

**Figure 13:**
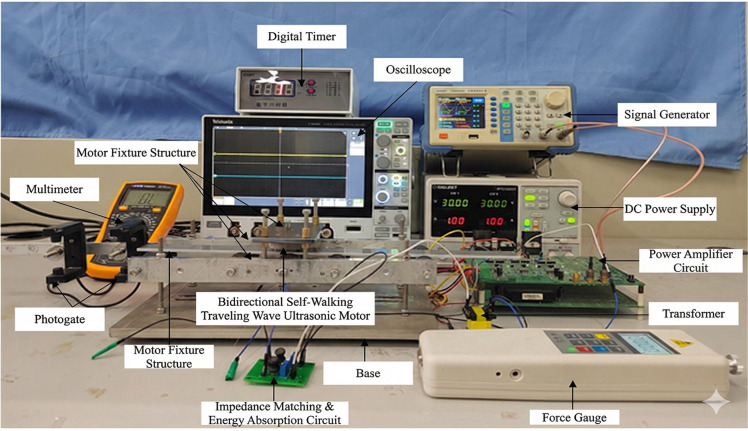
Experimental Platform of a Bidirectional Self-Propelled Traveling-Wave Linear Ultrasonic Motor.

The measurement instruments include a Digital Timer, Photogate, Force Gauge, Oscilloscope, and Multimeter. Among them, the Digital Timer and Photogate are mainly used to measure the velocity characteristics of the motor, the Force Gauge is employed to measure the linear blocking force, and the Multimeter is used to measure the resistance value of the sliding rheostat. The schematic diagram of the experimental platform is shown in Figure 13.

During the experiments, the excitation source consisted of two phase-adjustable sinusoidal AC signal generators. After power amplification, the two excitation signals were separately applied to the two piezoelectric ceramics at the excitation end of the stator. The two signals had identical amplitudes with a phase difference of 180°, so as to excite the desired bending traveling-wave mode in the stator. The excitation frequency was set near the resonant frequency of the motor, and the operating state of the motor was controlled by adjusting the output voltage amplitude.

**Figure 14:**
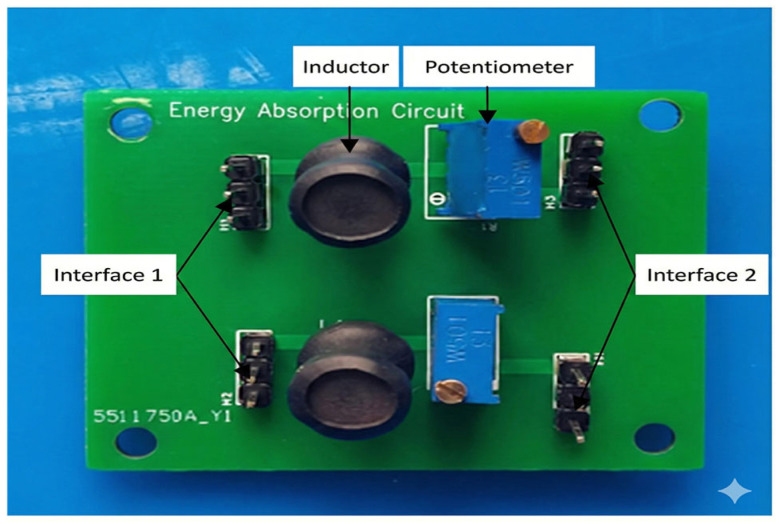
Impedance Matching and Energy Absorption Circuit of a Bidirectional Self-Propelled Traveling-Wave Linear Ultrasonic Motor.

At the opposite end of the stator, an external impedance matching and energy absorption circuit was connected to the piezoelectric ceramics to regulate the boundary conditions and reduce the reflected wave components during traveling-wave propagation, thereby increasing the proportion of the traveling-wave component inside the stator. In the experiments, the impedance matching circuit was composed of a series-connected inductor and resistor. The inductance value was kept constant, while the resistance value was varied to investigate its influence on the motor motion performance. The impedance matching and energy absorption circuit of the bidirectional self-propelled traveling-wave linear ultrasonic motor is shown in Figure 14.

The tested motor realizes self-propelled linear motion through contact between driving feet and the mover on the linear guide rail. Owing to the inherent flexibility of the adopted linear guide rail in the experimental system (Figure 13), an approximate 1 N preload is produced by its deformation. A photogate-digital timer system was used to measure the mover’s moving velocity during the experiments.

Based on the above experimental platform, the velocity characteristics of the motor were investigated under different excitation voltage amplitudes and impedance matching parameters, providing experimental support for subsequent performance analysis and structural optimization of the motor.

### 4.2. Prototype Demonstration

**Figure 15:**
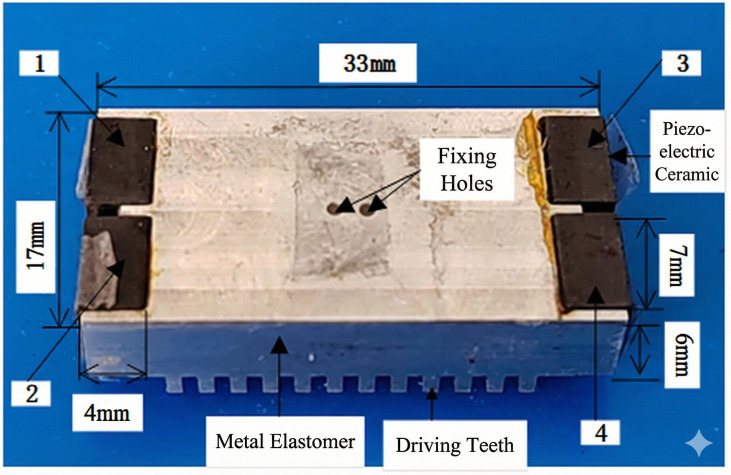
Photograph of the bidirectional self-propelled traveling-wave linear ultrasonic motor prototype.

Based on the model of the bidirectional self-propelled traveling-wave linear ultrasonic motor established in the finite element analysis, a three-dimensional mechanical model was developed using SolidWorks2023, and a physical prototype was subsequently fabricated, as illustrated in Figure 15.

A 7xxx-series aluminum alloy was selected as the material for both the metallic elastic body and the driving feet. PZT-4 was employed for the piezoelectric ceramics due to its favorable electromechanical coupling coefficient, yielding a piezoelectric strain constant d_15_ of up to 350 × 10^−12^ C/N in the torsional vibration mode.

### 4.3. Voltage–Speed Characteristics

**Figure 16:**
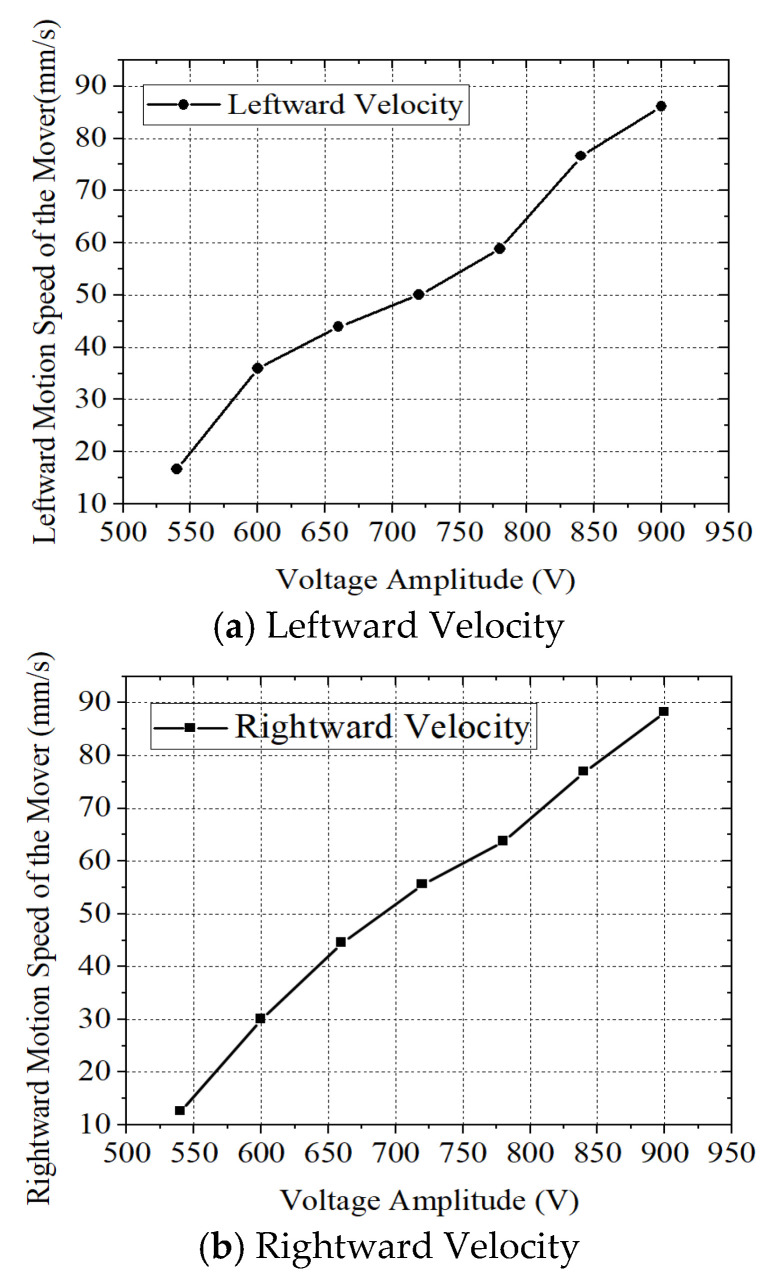
Voltage–Speed Characteristics.

In this experimental section, two piezoelectric ceramics at the excitation end were driven by high-frequency alternating voltages of equal amplitude with a 180° phase difference. The voltage amplitude was varied from 540 V to 900 V. The direction of motion toward the left was defined as positive, while that toward the right was negative. Multiple measurements were taken at each voltage level, and the average value was recorded as the final speed. The no-load speed of the motor as a function of voltage amplitude is shown in Figure 16.

According to the results shown in Figure 16, when the mover traveled in the forward direction, the maximum speed of 88.28 mm/s was achieved at a high-frequency voltage of 900 V, while the minimum speed of 12.65 mm/s was recorded at 540 V. In the reverse direction, the maximum speed reached 86.12 mm/s at 900 V, and the minimum speed was 16.69 mm/s at 540 V. As the voltage gradually increased, the mover’s speed increased correspondingly in both forward and reverse directions. Overall, the speeds in the forward and reverse motions were approximately equal, demonstrating the motor’s good structural symmetry. It can be observed that the velocity did not reach a clear saturation trend within the tested voltage range. In principle, further increasing the excitation voltage would increase the stator vibration amplitude and enlarge the elliptical motion of the driving feet, thereby enhancing the frictional driving force and increasing the motor velocity. However, such growth would not remain linear indefinitely due to nonlinear frictional behavior, contact saturation effects, and material damping. In this study, the maximum excitation voltage was limited to 900 V to ensure stable operation and structural safety of the prototype. Higher voltages would result in stronger electric fields in the piezoelectric ceramics and larger vibration amplitudes, potentially increasing dielectric stress and frictional wear at the contact interface. Therefore, 900 V was selected as a practical upper bound for safe and repeatable experimental operation.

### 4.4. Resistance Parameter–Speed Characteristics

**Figure 17:**
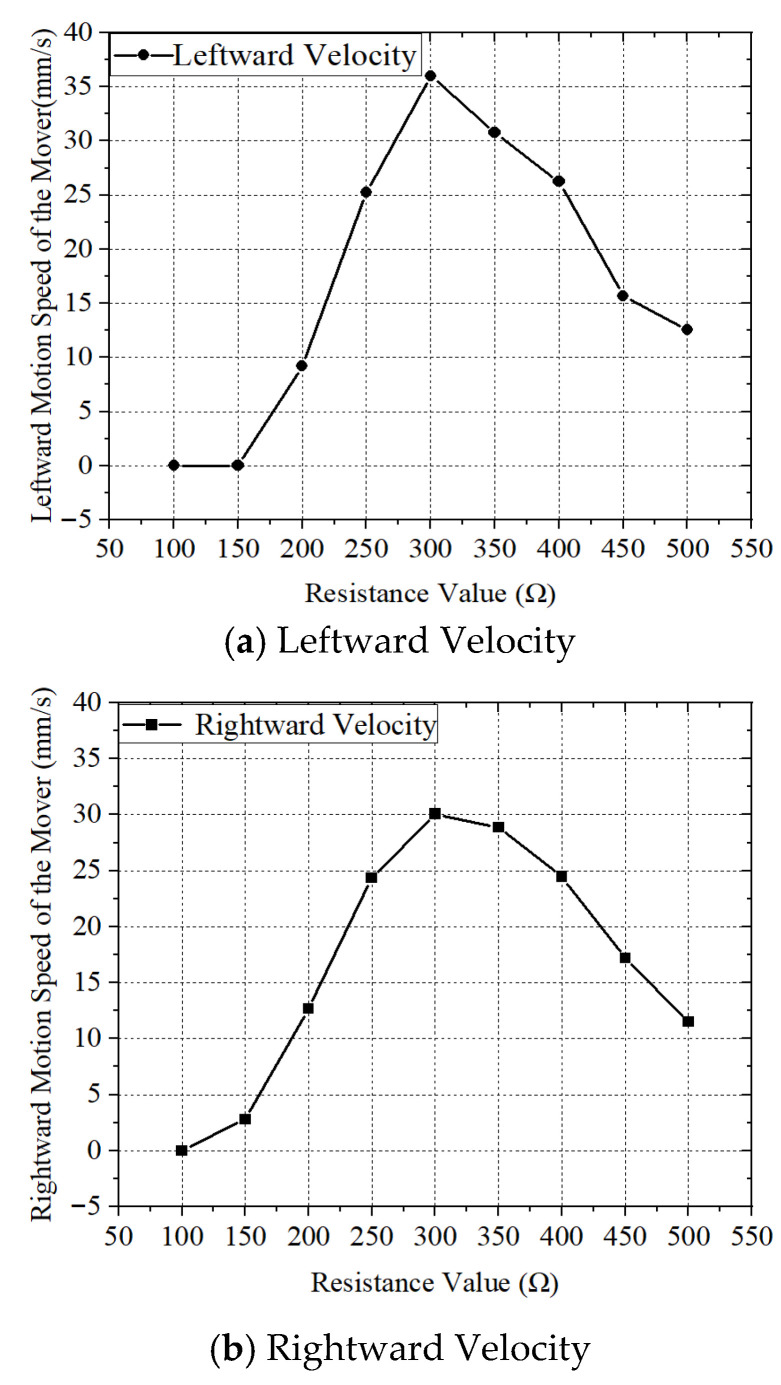
Resistance Parameter–Speed Characteristics.

Unlike conventional traveling-wave linear ultrasonic motors, which primarily adjust the input voltage to control the motor’s speed, the motor proposed in this study can vary the motor speed by adjusting the impedance parameters. The applied AC voltage amplitude and the inductance in the impedance matching circuit were kept fixed at 600 V and a constant inductance value, respectively, while the resistance parameter was varied from 100 Ω to 500 Ω. The no-load speeds of the motor at different resistance values obtained from the experiments are shown in Figure 17.

According to the results shown in Figure 17, at an applied voltage of 600 V, the maximum speed of the motor during forward motion was 30.06 mm/s when the resistance in the impedance matching circuit was 300 Ω, while the minimum speed was 0 mm/s at 100 Ω. For reverse motion under the same voltage, the maximum speed of 35.99 mm/s was also observed at 300 Ω, whereas speeds dropped to 0 mm/s at resistance values of 100 Ω and 150 Ω. It can be seen that the motor speed presents a distinct trend of rising first and then falling with the increase in resistance value, which fully reflects the critical role of impedance matching in the energy transmission and vibration excitation process of the ultrasonic motor. These results indicate that the optimal impedance matching resistance is approximately 300 Ω; increasing or decreasing the resistance from this value reduces the motor’s speed, as the impedance mismatch will lead to the reflection of electrical energy at the piezoelectric ceramic interface and the attenuation of effective vibration excitation energy of the stator.

### 4.5. Voltage–Blocking Force Test

**Figure 18:**
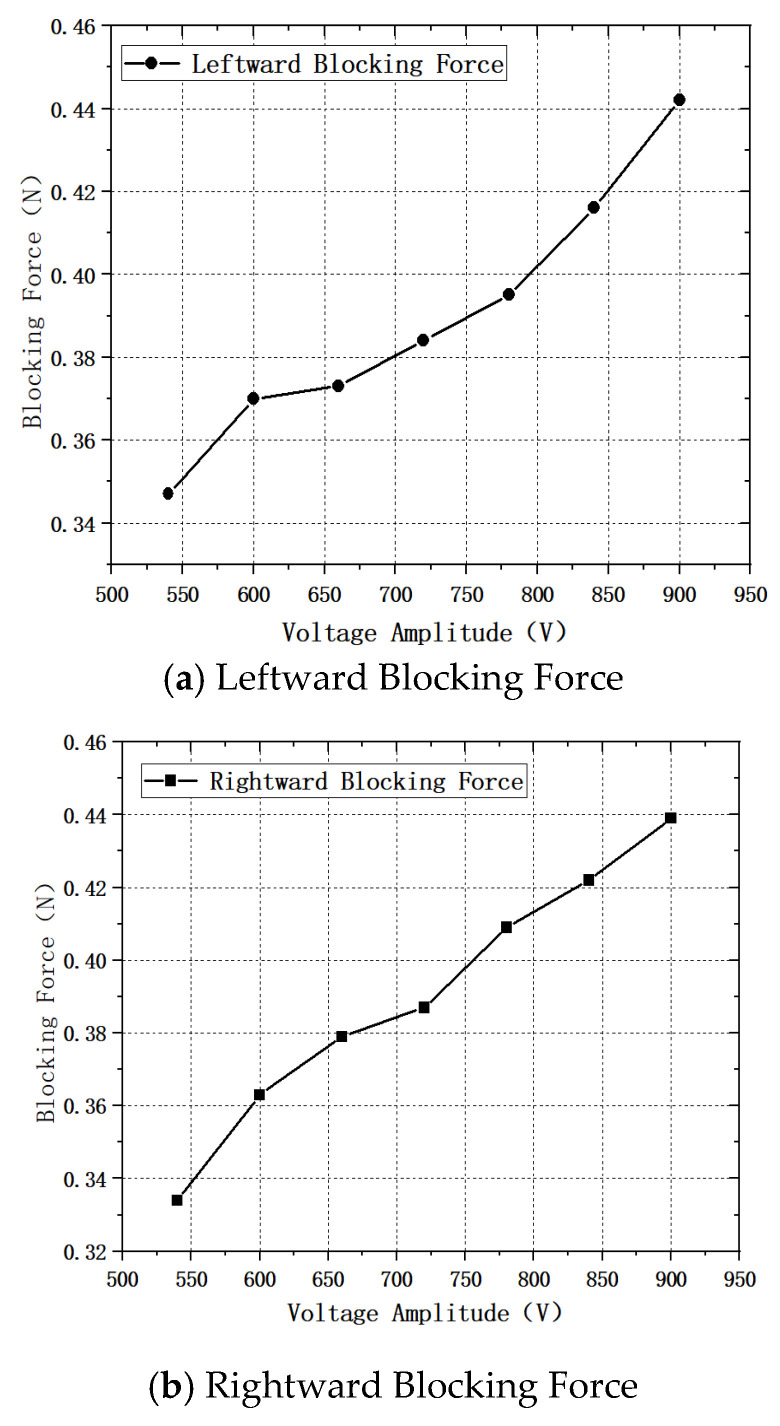
Voltage–Blocking Force Characteristics.

Following the procedure of the voltage–speed measurement test, in this experimental section a high-frequency AC voltage with equal amplitude and a phase difference of 180° was applied to the two piezoelectric ceramics at the excitation end. The voltage amplitude was varied from 540 V to 900 V, and the preload between the stator and the slider was kept constant during the whole test to ensure the consistency of the contact friction condition. The measured linear blocking force of the motor at different voltage amplitudes is shown in Figure 18.

According to the results shown in Figure 18, when the mover travels in the forward direction, the maximum linear blocking force reaches 0.439 N at a high-frequency voltage of 900 V, while the minimum value is 0.334 N at 540 V. When the mover travels in the reverse direction, the maximum linear blocking force is 0.442 N at 900 V, and the minimum value is 0.347 N at 540 V. As the voltage gradually increases, the linear blocking force of the mover increases in both the forward and reverse directions.

### 4.6. Impedance Parameter–Blocking Force Test

**Figure 19:**
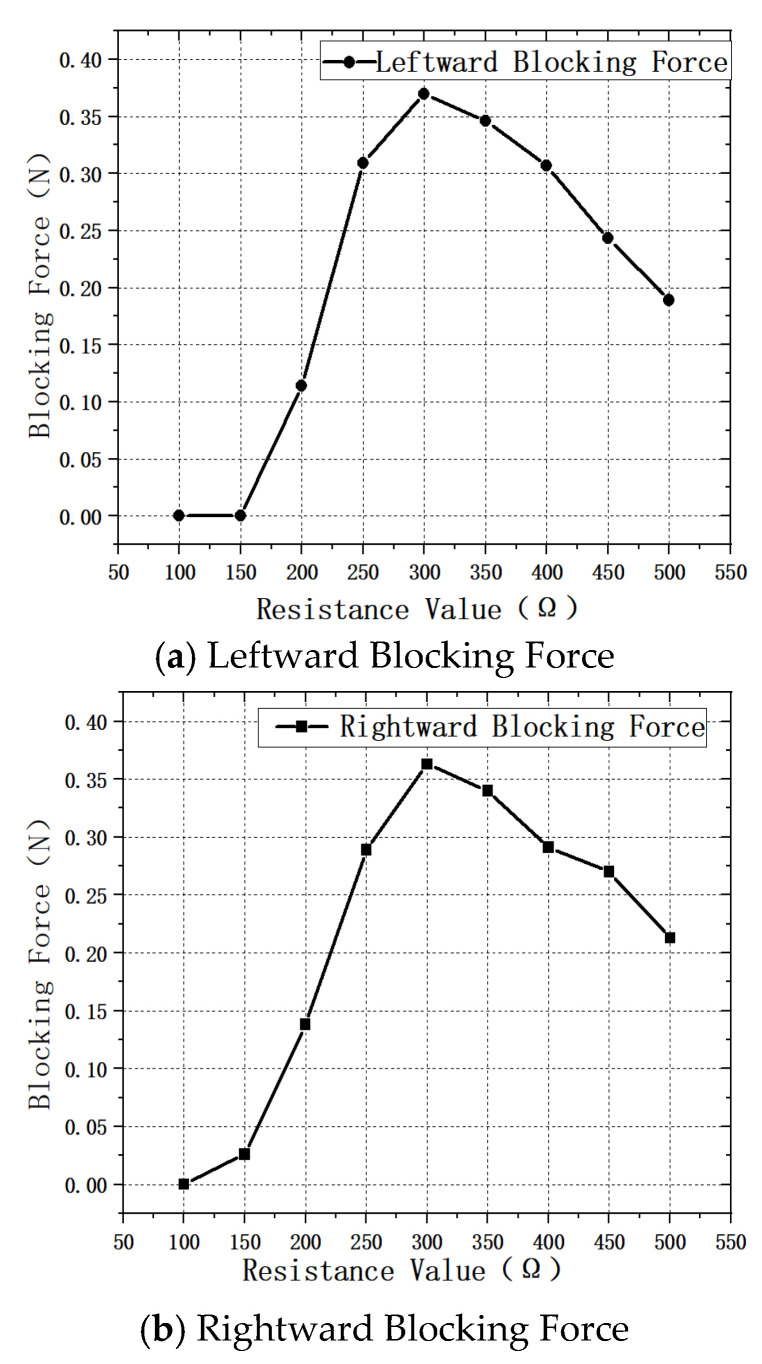
Impedance Parameter–Blocking Force Characteristics.

Referring to the experimental procedure used for measuring the impedance-parameter–speed characteristics, the linear blocking force of the motor is adjusted by varying the degree of impedance matching. In the impedance matching circuit, the resistance parameter is varied from 100 Ω to 500 Ω. The measured linear blocking force of the motor at different resistance values is shown in Figure 19.

According to the results shown in Figure 19, at an applied voltage of 600 V, the maximum linear blocking force during forward motion is 0.363 N when the resistance in the impedance matching circuit is 300 Ω, while the blocking force is 0 N at 100 Ω. Under the same voltage in the reverse direction, the maximum linear blocking force reaches 0.37 N at 300 Ω, and the minimum value of 0 N occurs at resistance values of 100 Ω and 150 Ω. These results confirm that the optimal resistance for impedance matching is approximately 300 Ω; increasing or decreasing the resistance from this value leads to a reduction in the linear blocking force of the motor.

## 5. Discussion

Existing studies on traveling-wave linear ultrasonic motors have explored end absorbers to suppress reflections and enhance traveling-wave quality [27,28]. Some works also focus on self-propulsion and high-thrust systems using longitudinal waves or dual-transducer configurations [29,30,31], while others have implemented bidirectional motion control for miniature systems [32]. It is undeniable that the proposed motor is slightly inferior to some high-power traveling-wave ultrasonic motors reported in the literature in terms of mechanical output performance such as rotational speed and blocking force. However, the core innovation of this work is not the pursuit of extreme output power, but the realization of the motor’s self-propelled function through a simplified boundary-free structure. Conventional traveling-wave linear ultrasonic motors usually have to rely on fixed boundary conditions, complex energy-absorbing systems and external clamping fixtures to generate stable traveling waves. Such auxiliary structures not only increase the complexity, weight and volume of the system, but also fundamentally restrict the motion form of the motor, confining it to operate only at a fixed position. In contrast, this study integrates the excitation and energy-absorbing piezoelectric ceramics into the protruding structures at both ends of the stator, thus successfully realizing the true self-propelled motion. Notably, in comparison with the high-thrust ultrasonic motor proposed by Wu et al. [21], although there is a certain gap in the absolute thrust value of the motor in this paper, the essence of this difference lies in the fundamental disparities in their design objectives and volume scales: the motor developed by Wu’s team achieves a maximum thrust of 24.5 N with a volume of 140 cm^3^, while the motor in this paper realizes a maximum thrust of 0.44 N with a compact volume of only 2.55 cm^3^. From the perspective of the core indicator of maximum thrust per unit volume, the performances of the two are basically on a par.

Simulation results reveal a non-uniform amplitude distribution along the stator length, reflecting the presence of a standing-wave component in the composite wave. However, the amplitude variation remains within a controllable range, confirming that the traveling-wave component dominates and can generate effective frictional driving. This observation aligns with experimental trends, where higher excitation voltages result in increased speed and blocking force, as larger excitation amplitudes correspond to larger elliptical motion at the contact interface, enhancing frictional drive capability.

Notably, impedance-matching tests show a clear “optimal value” in the matching resistance. When the impedance deviates from this optimal state, energy absorption weakens, reflections increase, and the traveling-wave proportion decreases, leading to degraded performance. This aligns with the conclusions drawn from the equivalent-circuit analysis, confirming that impedance adjustment offers an effective alternative to the traditional methods of voltage and frequency tuning.

Nevertheless, the current design still has some limitations. To further improve the motor’s performance, future research will focus on the following three aspects. Unlike traditional fixed traveling-wave linear ultrasonic motors, this motor can achieve self-propulsion without mechanical clamping. However, due to time constraints, we did not measure the output characteristics of the driving teeth on both sides under different excitation voltages or different impedance parameters at the absorbing end. In future work, a new mover structure will be designed to characterize the motor’s performance with varying parameters on both sides of the driving teeth. Second, the excitation/energy-absorbing effects on the front and rear sides of the stator are coupled. Due to time limitations, we did not conduct an in-depth analysis of the coupling effects on the motor’s performance, which led to insufficient theoretical support for the steering control strategy. Further research will explore this coupling effect in more detail based on extensive experimental data. Third, an integrated design of the stator and mover is required to optimize the contact characteristics between the motor and the mover, thereby simultaneously enhancing the motor’s output performance and operational stability.

## 6. Conclusions

This paper proposes and validates a bidirectional self-propelled traveling-wave linear ultrasonic motor, featuring boundary-condition regulation and impedance-matching energy absorption. Adopting a straight-beam stator structure, the motor achieves reversible control of the traveling wave propagation direction by switching the piezoelectric/inverse piezoelectric modes of the piezoelectric ceramics at both ends, thereby enabling stable bidirectional linear motion without mechanical clamping.

Finite element modeling and simulations—including modal analysis, harmonic response analysis, and driving tooth elliptical trajectory analysis—were systematically conducted to verify the rationality and feasibility of the structural design. The simulation results confirm that the stator vibrates in the desired B(3,1) bending mode, with the traveling-wave component dominating the composite waveform (maximum-minimum amplitude difference of 0.13 μm) and generating effective frictional driving force through regular elliptical motion of the driving teeth.

Experimental tests on a dedicated platform demonstrate the motor’s excellent performance: under a 900 V excitation voltage, the maximum forward no-load speed reaches 88.28 mm/s and the maximum reverse speed is 86.12 mm/s, reflecting good structural symmetry. At an excitation voltage of 600 V, the optimal impedance matching resistance is approximately 300 Ω, at which the motor achieves a maximum linear blocking force of 0.37 N (reverse direction) and 0.363 N (forward direction). Notably, impedance parameter adjustment provides an effective alternative to traditional voltage/frequency tuning for speed and blocking-force regulation, highlighting the motor’s flexible control capability.

With its compact structure, rapid dynamic response (vibration initiation within 0.146 μs), and adjustable speed and direction, the proposed motor is promising for precision drive and positioning applications in microelectronics, biomedical devices, and miniature robotic systems. This study provides a novel design paradigm for streamlined, self-propelled ultrasonic motors and lays a foundation for further optimization of contact characteristics and steering control strategies.

## Figures and Tables

**Table 1:** Structural Parameters of the Bidirectional Self-Propelled Traveling-Wave Linear Ultrasonic Motor.

Location		Length (mm)	Width (mm)	Height (mm)
metal elastomer		25	17	6
protruding structure	both sides middle section	4 2	1 1	6 6
piezoelectric ceramic		4	7	6
driving foot		1	2	2

## Data Availability

The original contributions presented in this study are included in this article. Further inquiries can be directed to the corresponding author.
